# Structure, Properties and Degradation of Self-Assembled Fibrinogen Nanofiber Scaffolds

**DOI:** 10.1021/acsabm.4c00761

**Published:** 2024-09-03

**Authors:** Till Strunk, Arundhati Joshi, Mahta Moeinkhah, Timon Renzelmann, Lea Dierker, Dietmar Grotheer, Nina Graupner, Jörg Müssig, Dorothea Brüggemann

**Affiliations:** † Institute for Biophysics, University of Bremen, Otto-Hahn-Allee 1, 28359 Bremen, Germany; ‡ Hochschule Bremen − City University of Applied Sciences, Neustadtswall 30, 28199 Bremen, Germany; § Chemical Process Engineering, Faculty of Production Engineering, University of Bremen, Leobener Str. 6, 28359 Bremen, Germany; ∥ HSB − City University of Applied Sciences, Department of Biomimetics, The Biological Materials Group, Neustadtswall 30, 28199 Bremen, Germany; ⊥ MAPEX Center for Materials and Processes, University of Bremen, 28359 Bremen, Germany

**Keywords:** skin substitutes, self-assembly, wound healing, mechanical properties, enzymatic degradation, biomimetics

## Abstract

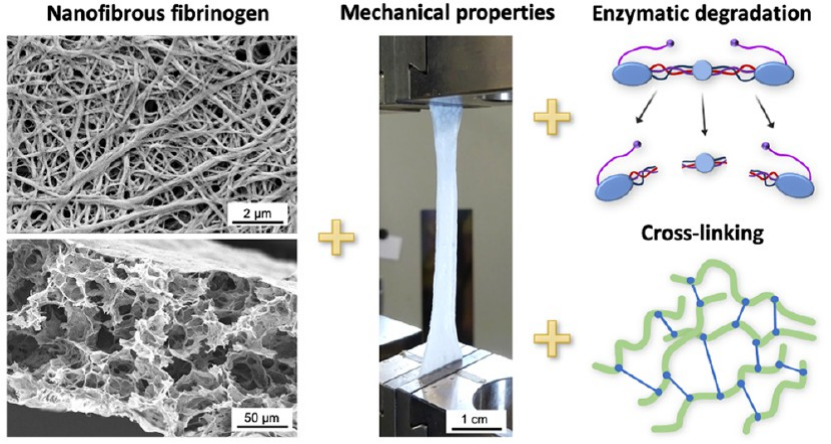

Self-assembled fibrinogen nanofibers are promising candidates for skin tissue engineering due to their biocompatibility and ability to mimic the native blood clot architecture. Here, we studied the structure–property relationship and degradation of rehydrated fibrinogen nanofibers prepared by salt-induced self-assembly, focusing on the effect of scaffold layering, cross-linking time and freeze-drying. Optimal fiber stability was achieved with cross-linking by formaldehyde (FA) vapor, while treatment with liquid aldehydes, genipin, EDC, and transglutaminase failed to preserve the nanofibrous architecture upon rehydration. Scaffold layering did not significantly influence the mechanical properties but changed the scaffold architecture, with bulk fiber scaffolds being more compact than layered scaffolds. Freeze-drying maintained the mechanical properties and interconnected pore network with average pore diameters around 20 μm, which will enhance the storage stability of self-assembled fibrinogen scaffolds. Varying cross-linking times altered the scaffold mechanics without affecting the swelling behavior, indicating that scaffold hydration can be controlled independently of the mechanical characteristics. Cross-linking times of 240 min increased scaffold stiffness and decreased elongation, while 30 min resulted in mechanical properties similar to native skin. Cross-linking for 120 min was found to reduce scaffold degradation by various enzymes in comparison to 60 min. Overall, after 35 days of incubation, plasmin and a combination of urokinase and plasminogen exhibited the strongest degradative effect, with nanofibers being more susceptible to enzymatic degradation than planar fibrinogen due to their higher specific surface area. Based on these results, self-assembled fibrinogen fiber scaffolds show great potential for future applications in soft tissue engineering that require controlled structure–function relationships and degradation characteristics.

## 1 Introduction

Skin regeneration is a major challenge in wound healing, affecting nearly 40 million people globally,^1^ demanding innovative strategies for skin tissue engineering.^2^ Tissue-engineered substitutes, particularly cell-free scaffolds, offer promising alternatives to the traditional use of auto- and allografts that often suffer from limited availability and time-consuming precultivation.^2−4^ These scaffolds need to possess a porous architecture for gas exchange and cell infiltration, have suitable mechanical properties, good swelling capacity to absorb exudates, and should have tailorable degradation properties to facilitate optimal wound repair.^2,5−7^

Fiber-based materials, resembling the porous network in the extracellular matrix (ECM) of native skin, are ideal candidates for such scaffolds.^4^ While fiber scaffolds from synthetic polymers lack important cell binding sites and have slow degradation rates, those from biopolymers like collagen or chitosan often exhibit low mechanical strength, deformation and shrinkage in aqueous environment.^2,5^ In particular, mimicking the mechanical properties of native skin is a major challenge in skin tissue engineering, as they vary strongly depending on the measurement technique or the anatomical site, age and gender,^8−12^ which has prompted research into the development of alternative scaffold materials.^4,7,13^

After blood coagulation, a nanofibrous fibrin clot closes the wound and functions as a provisional ECM that enables integrin-mediated cell adhesion during the initial wound healing stage.^14^ In the subsequent proliferation phase, various cells deposit fibronectin, elastin, collagen and other ECM proteins, restoring the structural integrity and mechanical properties of native skin tissue.^14^ Fibrin and its precursor fibrinogen are therefore widely used as candidates for fibrous scaffolds to mimic the architecture of native blood clots and to serve as provisional ECM in skin tissue engineering.^15,16^ However, fibrin, often used as a fibrous hydrogel, has several drawbacks that make it unsuitable for many biomedical applications, such as rapid degradation, structural fragility and shrinkage, and poor mechanical properties.^2,16,17^ Moreover, the production of fibrin(ogen) hydrogels may require the addition of polymers, enzymes or molecular modifications to obtain mechanically stable scaffolds with adjustable degradation profiles, which would significantly increase production time and cost.^15,17^ On the other hand, electrospun fibrinogen nanofibers can mimic the native blood clot architecture and were found to support the growth of fibroblasts and endothelial cells,^18−21^ which are both important in wound healing.^4^ Although the mechanical properties and degradation of electrospun fibrinogen can be well adjusted with different cross-linkers,^19,20,22^ this process requires high protein concentrations of up to 200 mg/mL, which increases scaffold fabrication costs.^21,23^ Swelling-related porosity reduction in aqueous media is another disadvantage of electrospun fibrinogen that would limit cell infiltration during skin repair^4,20,22,23^ and makes this scaffold material less suitable for skin tissue engineering.

To meet the need for mechanically strong biopolymer scaffolds with porous architecture and controllable degradation for skin tissue engineering, salt-induced self-assembly of fibrinogen could be a promising strategy.^24,25^ By adding different types of salts to an aqueous fibrinogen solution and drying under controlled conditions, this method produces dense nanofibers with fiber diameters between 100 and 300 nm.^24^ Interestingly, only monovalent salts induce nanofiber formation, while divalent salts result in smooth fibrinogen precipitates,^26^ which are therefore not suited as wound healing scaffolds. Fiber assembly of fibrinogen is accompanied by mild secondary structure changes, yet without inducing any pathogenic amyloid transitions.^25^ These changes are even reversed to a conformationally more native state when a post-treatment with formaldehyde vapor is introduced to preserve the fiber architecture, followed by hydration.^25^ So far, self-assembled fibrinogen nanofibers supported the proliferation and migration of keratinocytes and different fibroblast types in both mono- and coculture.^27,28^ Moreover, they enhanced spreading of blood platelets, minimized their procoagulant activity^29^ and could prevent infiltration with *E. coli* bacteria,^27^ which makes them highly biocompatible. Therefore, this study will provide a fundamental understanding of the structure–function relationship and degradation characteristics of self-assembled fibrinogen nanofibers to evaluate their suitability as degradable scaffold material for wound healing and skin tissue engineering.

## 2 Experimental Section

### 2.1 Fibrinogen Solutions

Fibrinogen nanofibers were prepared with salt-induced self-assembly using 100% clottable fibrinogen (Prod. no. 341556, Merck, Darmstadt, Germany).^24,25^ Fibrinogen was dissolved in 10 mM NH_4_HCO_3_ and dialyzed against 10 mM NH_4_HCO_3_ (Carl Roth, Karlsruhe, Germany) overnight with 14 kDa cutoff cellulose membrane dialysis tubing (Sigma, Steinheim, Germany). This step was necessary to remove any residual salts from the manufacturing process, that would hinder the salt-induced self-assembly of nanofibers. For subsequent experiments, the protein concentration was adjusted between 2.5 and 12 mg/mL (see Supporting Information, Table S1). All solutions were prepared with Milli-Q water from a TKA water purification system (Thermo Fisher Scientific, Schwerte, Germany).

### 2.2 Self-Assembly of Nanofibrous Fibrinogen Scaffolds for Mechanical Testing

For mechanical testing, reusable molds were created using a dog-bone-shaped PVC specimen (KTK Kunststofftechnik Vertriebs GmbH, Germering, Germany) with previously published dimensions.^27^ Polydimethylsiloxane (PDMS) molds were made by mixing base (A) and curing agent (B) from a SYLGARD 184 Silicone Elastomer Kit (Distrelec GmbH, Bremen, Germany) in a 10:1 ratio, followed by 24 h curing at 60 °C in an oven (Memmert GmbH + Co. KG, Schwabach, Germany). After removing the PVC shapes, the PDMS molds were filled with fibrinogen solutions ranging from 2.5 to 12 mg/mL and dried with varying volumes and concentrations of phosphate-buffered saline (PBS, Thermo Fisher, pH 7.4; see Supporting Information, Table S1). Nanofibrous fibrinogen scaffolds were dried for 24 h at 25 °C and 30% relative humidity in a custom-built climate chamber. To obtain scaffolds with six layers and a total protein mass of 15 or 30 mg, this process was repeated five times. Single-layer fibrinogen scaffolds (bulk samples) with the same mass were prepared in one step by increasing the concentrations, and 15 mg scaffolds with three layers were also prepared. Subsequently, cross-linking in 37% formaldehyde (FA, AppliChem GmbH, Darmstadt, Germany) vapor was performed for 120 min in a parafilm-sealed beaker, followed by 60 min of washing in Milli-Q water.^27^ In addition, cross-linking times of 30, 120, and 240 min were studied for three-layer samples with 15 mg fibrinogen. Fibrin references with 15 mg fibrinogen were prepared as single- or three-layer samples using 40 U/ml thrombin (Sigma) or 10 U/ml thrombin in PBS and did not undergo FA vapor cross-linking.

### 2.3 Scaffold Preparation for Enzymatic Degradation

For enzymatic degradation studies, 15 mm glass coverslips (VWR, Darmstadt, Germany) were cleaned with piranha solution (3:1 of 95% sulfuric acid (H_2_SO_4_): 30% hydrogen peroxide (H_2_O_2_)) and treated with 5% (3-aminopropyl)triethoxysilane (APTES, Sigma) in ethanol (C_2_H_5_OH). To enable upscaling of the fibrinogen scaffold dimensions and thickness for future applications as a degradable wound dressing, we have re-evaluated our previous routine for the dialysis of fibrinogen.^24^ This led to an adjustment of our standard concentration for fiber assembly by a factor of 2. Therefore, 2.5 mg/mL fibrinogen corresponds to our previously published concentration of 5 mg/mL. To obtain nanofibrous and planar fibrinogen scaffolds in this work, 3 mg/mL fibrinogen along with either 2.5x PBS, pH 7.4 or 5 mM NH_4_HCO_3_ were dried on APTES-modified coverslips at a 30% relative humidity and 25 °C for 12 h. Planar fibrinogen was used as a reference substrate with smooth surface topography^24^ to study the effects of the 15-fold higher surface roughness of nanofibrous scaffolds on enzymatic degradation. Fibrous and planar fibrinogen scaffolds were subjected to either 60 or 120 min of cross-linking in FA vapor. After devaporization for 30 min, all samples were washed three times 15 min each with deionized water. Prior to long-term degradation, all fibrinogen scaffolds were placed in wells of not treated Corning Costar 24-well plates (Sigma) and sterilized for 30 min using the UV light of a laminar flow cabinet (ESI Flufrance).

### 2.4 Cross-Linking of Self-Assembled Fibrinogen Nanofibers

A post-treatment of self-assembled fibrinogen scaffolds through cross-linking is necessary to maintain the nanofibrous architecture, which would otherwise dissolve in aqueous media.^30^ Therefore, to study the effect of different cross-linkers on fiber stability in aqueous environment, fibrinogen nanofibers were assembled on ethanol-cleaned glasses (30 min submerged sonication) using the same settings as for degradation studies. Different cross-linking methods were selected based on previous studies using synthetic cross-linkers for electrospun fibrinogen^20^ or more native transglutaminase 2, which is found in tissues and erythrocytes, for fibrinogen films.^31^ We used 200 μL of 50 mM 1-ethyl-3-(3-(dimethylamino)propyl) carbodiimide (EDC) (Sigma) in Milli-Q water and covered the scaffolds for 30 min. For transglutaminase 2 (TG2), thawed aliquots were combined with Tris buffer to a final concentration of 0.995 mM TG2 (hTG2- T022, ZEDIRA, Darmstadt, Germany) in 50 mM Tris, 10 mM CaCl_2_ (pH 7.5). Scaffolds were covered with 200 μL TG2 for 60 min at 37 °C on a heating plate (IKA, Staufen, Germany). Alternatively, fibrinogen nanofibers were incubated in 200 μL of 30 mM genipin (Sigma) for 60 min or in 200 μL 2% (v/v) glutaraldehyde (GA; Applichem GmbH, Darmstadt, Germany) or 4% (v/v) FA for 30 min. For vapor fixation, 1 μL of 50% GA aldehyde solution per cm^3^ was filled into a Petri dish. GA solution and the samples were placed in a sealed box for 120 min. Residual cross-linkers were removed by three washing steps in 2 mL Milli-Q water for 5 min, followed by drying. Our previous FA vapor cross-linking routine^27^ was used as reference treatment. Control samples were not cross-linked and analyzed with and without subsequent washing.

### 2.5 Mechanical Testing

Tensile testing of hydrated fibrinogen scaffolds for Young’s modulus, tensile strength, elongation at break, and toughness at maximum stress was conducted using a Zwick Z020 universal testing machine (Zwick/Roell GmbH, Ulm, Germany) equipped with a 500 N load cell following our previous routine (*n* = 7 to *n* = 12).^27^ To remove scaffolds from the PDMS molds, they were immersed in Milli-Q water for at least 60 min before testing. Testing was performed at an elongation rate of 10 mm/min, and the Young’s modulus was calculated from the linear-elastic region in the range between 2 and 7% elongation by linear regression of the stress–strain curves. All samples were 8 mm wide, and sample thickness was measured with a caliper with 1 μm accuracy after each test to prevent sample damage prior to testing. Additionally, tensile testing of 15 mg freeze-dried bulk scaffolds (*n* = 7) was carried out. Due to the small sample size, tensile tests were performed with strip-like samples with dimensions of 30 mm × 8 mm at a speed of 10 mm/min and a clamping length of 15 mm. The test specimen thickness was comparable to the air-dried and rehydrated samples (FG 1L 15 mg) with a median value of 60 ± 10 μm.

We measured the bending stiffness of rectangular fibrinogen scaffolds (16 mm × 32 mm) in a home-built setup after rehydration in Milli-Q water. Scaffolds were placed over the edge of a horizontally positioned microscope slide (VWR) and excess water was removed. Side view images of the overlaying edge were taken with an iPhone 11 camera (Apple, Cupertino, USA). Images were analyzed with ImageJ (NIH) to calculate the scaffold bending stiffness, according to Wei.^32^

For statistical analysis with R (RStudio version 1.3.1093, Boston, USA), a Shapiro-Wilk and Levene test for normal distribution and variance equality was conducted. Subsequently, a one-way ANOVA test with Tukey’s test or Kruskal–Wallis with pairwise Wilcoxon test was performed with p-value adjustment after Bonferroni based on distribution and variance assumptions (α = 0.05). Significant differences were represented by letters above box-whisker plots, where the bold black bar indicates the median and the box’s ends show the first and third quartiles. Whiskers indicate the data range unless a data point exceeds ≥1.5 times the interquartile range (outlier).

### 2.6 Swelling Characteristics

To study the effect of water uptake, 15 mg fibrinogen scaffolds were used that were cross-linked for 60 and 120 min, respectively. Calipers with 1 μm accuracy were employed for initial thickness measurements that were compared with optical analysis using an Axiovert 40 CFL inverted microscope at 40× magnification (Carl Zeiss, Göttingen, Germany). For upright scaffold mounting, a sample holder was 3D-printed from a thermoplastic PLA filament with a Makerbot Replicator fifth Generation (Stratasys GmbH, Rheinmünster, Germany). Thickness measurements were obtained from iPhone 11 images of magnified scaffold edges, ensuring reproducible distances with a 3D-printed PLA holder (see Supporting Information, Figure S1). Image analysis using ImageJ and reference images from a calibration slide was conducted.

For mass change analysis of rehydrated fibrinogen scaffolds, a VCL4003 humidity chamber (Vötsch Industrietechnik GmbH, Reiskirchen, Germany) and an analytical balance (ABT 120–5DM, Kern & Sohn GmbH, Balingen, Germany) were used. Mass was recorded at 0%, 65%, and 85% relative humidity. Dry mass (0%) was determined after 6 h at 60 °C, while samples at 65% and 85% humidity were conditioned at 20 °C for at least 18 h.

### 2.7 Long-Term Degradation of Fibrinogen Scaffolds

To study the influence of various serine proteases on the stability of FA vapor-cross-linked fibrinogen scaffolds, enzyme solutions were prepared in HEPES buffered saline. HEPES buffer contained 10 mM 2-(4-(2-Hydroxyethyl)-1-piperazinyl)-ethansulfonic acid (Thermo Fisher), 150 mM NaCl (VWR) and 5 mM CaCl_2_ (Sigma) in deionized water and was adjusted to pH 7.4 with NaOH (VWR). Final enzyme concentrations for long-term scaffold degradation were: 1 U/mL of thrombin from bovine plasma (Sigma), 0.01 U/mL plasmin from human plasma (Sigma), 1 μg/mL urokinase-type plasminogen activator (Merck, Darmstadt, Germany), 0.01 U/mL plasminogen from human plasma (Sigma), and a mixture of plasminogen and urokinase.

Subsequently, UV-sterilized scaffolds in 24-well plates were incubated in 2 mL/well of each enzyme solution at 37 °C in an incubator (Heracell, Thermo Fisher) for 35 days. Fibrinogen released into the supernatant was analyzed every 7 days using UV–vis spectroscopy in a Multiskan Sky microplate spectrophotometer (Thermo Fisher). For this purpose, 50 μL of the supernatant solution was pipetted into the wells of a 384-well UV-Star microtiter plate (Greiner Bio-One, Frickenhausen, Germany), and the absorbance was measured at 280 nm. To analyze the basal degradation in the absence of enzymes, scaffolds were incubated with HEPES buffered saline as negative controls. The corresponding absorbance values were treated as blanks and subtracted from the readings of scaffolds incubated in enzymes.

The released fibrinogen concentration was calculated using the extinction coefficient 1.51 mL·mg^–1^·cm^–1^ provided by Sigma. To account for evaporation and weekly supernatant removal, volume control experiments were conducted, for which 2 mL HEPES buffered saline per well without enzymes or scaffolds were incubated for the length of the experimental period. The volume was measured on each day of analysis. Finally, the total amount of fibrinogen released into the solution was calculated by multiplying the concentration of released fibrinogen with the amount of buffer left on each day of analysis. Three independent experiments (*n* = 3) were performed in triplicates for each scaffold and enzyme type, with statistical analysis conducted using Graphpad Prism 8 (GraphPad, San Diego, CA, USA).

To assess the molecular size of the degraded products, the solution collected after 35 days also underwent sodium dodecyl sulfate-polyacrylamide gel electrophoresis (SDS-PAGE). Briefly, 18 μL of each sample were mixed with NuPAGE LDS nonreducing sample buffer (Thermo Fisher) and denatured at 70 °C for 10 min in an Eppendorf Thermomixer (Webers GmbH, Oberhausen, Germany). Twenty μL/well of the denatured protein samples and 10 μL/well of HiMark prestained protein standard were loaded into the wells of a NuPAGE Tris-Acetate Mini Gel using NuPAGE Tris-Acetate SDS Running Buffer (all Thermo Fisher). Gels ran for 60 min at 150 V using a PowerEase Touch 120W Power Supply (230 VAC, Thermo Fisher). All gels were immersed in a 0.1% (w/v) Coomassie brilliant blue (Sigma) solution in 40% (v/v) methanol (VWR), 10% (v/v) acetic acid (Sigma) and 50% (v/v) deionized water on a shaker for 60 min at RT. After washing twice for 1 h each in 40% methanol, 10% acetic acid and 50% deionized water on the shaker at room temperature, the gels were placed in deionized water and photographed. The molecular weight of the protein sample bands was calculated by comparison to a standard curve obtained from a known protein standard ladder (Thermo Fisher) and relative migration distances. Three independent gels (*n* = 3) for each scaffold and enzyme type were analyzed, with representative gel images shown.

### 2.8 Scanning Electron Microscopy

To study the influence of layering on nanofiber porosity, we freeze-dried 15 mg samples with one or three layers (16 mm × 32 mm) using deep-freezing for 22 h in a laboratory system (Martin CHRIST GmbH) at 0.046 mbar and −50 °C ice condenser temperature. Before scanning electron microscopy (SEM) analysis, all samples were sputter-coated with approximately 7 nm gold, either in a Bal-Tec SCD 005 (Bal-Tec, Liechtenstein) or an EM ACE600 device (Leica Microsystems, Wetzlar, Germany). SEM analysis was performed with a JSM 6510 (Jeol, Eching, Germany) at 10 kV using a secondary electron detector. Pore sizes of freeze-dried scaffolds were statistically analyzed with ImageJ using the plugin BoneJ.^33^ To study the effect of enzymatic degradation on scaffold topography after 35 days, all scaffolds were air-dried and sputter-coated with gold for 25 s in a 108-auto system (Tecan GmbH, Dortmund, Germany). Subsequent SEM analysis was performed with a Zeiss Supra 40 device at 3 kV using an SE2 detector (Carl Zeiss AG, Oberkochen, Germany).

## 3 Results and Discussion

### 3.1 Influence of Different Cross-Linkers on the Morphology of Fibrinogen Nanofiber Scaffolds

**Figure 1:**
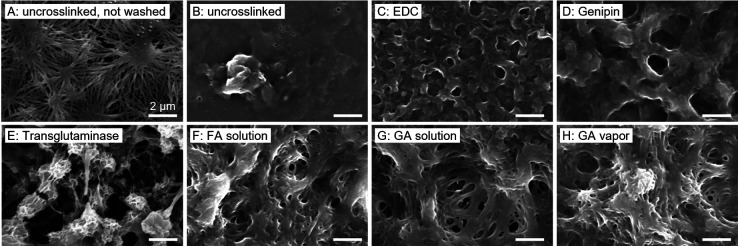
**Effect of different cross-linkers on fibrinogen fiber preservation after washing and drying.** No cross-linker was applied to the controls that were (A) not washed, or (B) washed with Milli-Q water. All other nanofibrous fibrinogen scaffolds were exposed to different cross-linkers: (C) EDC, (D) genipin, (E) transglutaminase, (F) FA solution, (G) GA solution, (H) GA vapor. The topographical differences found by SEM analysis show that a vapor cross-linker is best suited to preserve the porous fiber architecture upon rehydration, whereas this is not the case with aqueous cross-linkers.

Different cross-linkers like genipin, EDC and GA vapor were previously used to stabilize electrospun fibrinogen for cell culture studies, to enhance its mechanical characteristics and to slow degradation.^19,20,22^ Therefore, we analyzed the influence of various cross-linkers on the durability of self-assembled fibrinogen scaffolds in aqueous environment. Fibrinogen fibers that were washed without cross-linking dissolved completely, leaving only a planar, smooth surface or clots (see Figure 1B). This observation is consistent with our previous report^24^ and similar to the dissolution of electrospun gelatin fibers that were found to hydrolyze when no cross-linking was applied.^34^ After washing of fibrinogen nanofibers that had been cross-linked with EDC or genipin, hardly any fibers were observed anymore, and only a molten-like topography remained (see Figure 1C and D). When both cross-linkers were previously used with electrospun fibrinogen, they resulted in reduced cell migration due to increased scaffold stiffness and masking of potential cell-integrin binding sites.^20^ Moreover, EDC and genipin treatment caused cleavage of disulfide bonds under physiological conditions in various biopolymer films, resulting in their disintegration.^35^ Based on our findings and these previous reports we conclude that EDC and genipin are not suited for cross-linking of self-assembled fibrinogen nanofibers to be used in biomedical applications.

Transglutaminase is an enzyme that is often employed as a biological, nontoxic cross-linker under mild reaction conditions.^36^ However, the treatment of fibrinogen nanofibers with transglutaminase resulted in a completely different structure of nodules exhibiting a porous, spongy topography (see Figure 1E), making it impractical for our scaffolds. Since cross-linking of protein-based biomaterials with aldehydes is relatively inexpensive and simple due to rapid reactions with amino groups,^35^ we also compared FA and GA treatment. Cross-linking with FA and GA solutions for 30 min or GA vapor for 120 min preserved the fiber topography slightly with some fibers being visible (see Figure 1F, G, and H). Yet, redissolved fibers that merged into bundles with some gaps in the scaffold were observed in many parts of the surface. Overall, all cross-linking treatments were not as efficient as our previously established 120 min FA vapor treatment, where multiple layers of very well-defined individual fibers were visible (c.f. Figure 7A and 7H). In addition, we previously observed very good biocompatibility of fibrinogen nanofibers cross-linked with FA vapor with different cell types^27−29^ and therefore used this cross-linker to study its influence on the mechanical characteristics and degradation of self-assembled fibrinogen scaffolds.

### 3.2 Effect of Cross-Linking Time on the Scaffold Mechanics and Swelling

When we varied the FA cross-linking time of three-layered scaffolds with 15 mg fibrinogen, we obtained an average thickness of 41 to 48 μm (see Supporting Information, Table S2). The fibrin reference had the highest thickness of 52 μm, which might be due to thrombin-mediated cross-linking instead of FA vapor treatment. Interestingly, no clear trend based on cross-linking time was found for the thickness of nanofibrous fibrinogen scaffolds.

**Figure 2:**
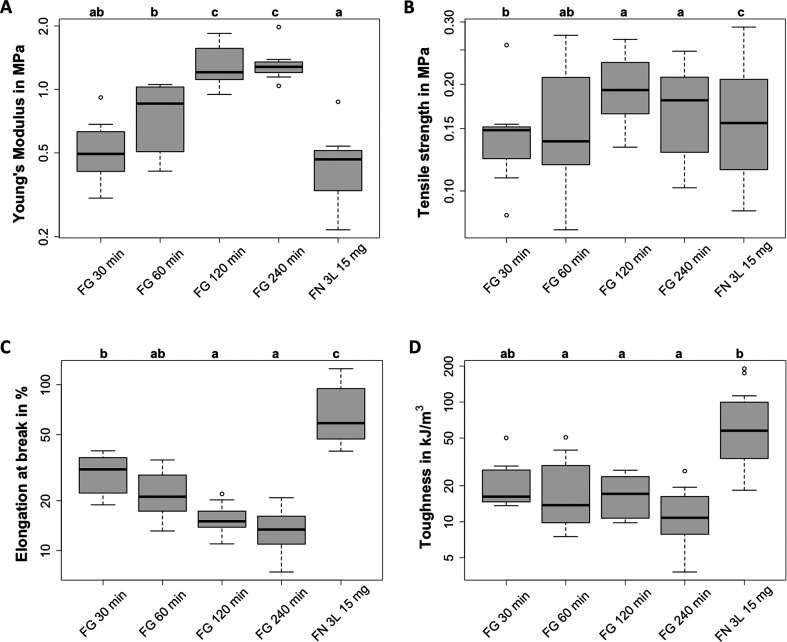
**Effect of cross-linking time on the scaffold mechanics.** Mechanical characteristics of fibrinogen (FG) and fibrin (FN) scaffolds with protein amounts of 15 mg deposited in three layers and cross-linked between 30 and 240 min. All data are presented on a logarithmic *y*-axis: (A) Young’s modulus, (B) tensile strength, (C) elongation at break, and (D) toughness. The sample size varied from *n* = 7 (FG 30 min and FG 240 min), *n* = 8 (FG 60 min), *n* = 9 (FG 120 min) to *n* = 12 (FN 3L 30 mg). Significant differences between sample parameters are indicated by different letters above the boxplot. Samples sharing at least one letter do not differ significantly from each other. In subfigure (B), no significant differences were found.

Previously, increasing cross-linking times enhanced the stiffness of electrospun fibrinogen.^20,22^ Here, we found the same trend for self-assembled fibrinogen nanofibers with an increase of the median Young’s modulus from 0.54 to 1.34 MPa when the cross-linking time was increased from 30 to 240 min (see Figure 2A). These values align well with native skin stiffness^7^ and are slightly below electrospun fibrinogen nanofibers treated with different cross-linking agents.^20,22^ A comparison of three-layer fibrin scaffolds without cross-linking to 30 min cross-linked fibrinogen scaffolds yielded a comparable median Young’s modulus of 0.45 MPa. This indicates that shorter cross-linking times yield mechanical properties similar to native fibrin, which is crucial for the mechanoregulation of wound healing.^37,38^

The tensile strength of fibrinogen nanofibers increased from about 0.15 MPa for 30 min to 0.20 MPa for 120 min and decreased to 0.17 MPa for 240 min cross-linking time (see Figure 2B). Since no significant differences were found and fibrin scaffolds also yielded a median tensile strength of 0.17 MPa we conclude that cross-linking time did not affect the tensile strength of self-assembled fibrinogen. Interestingly, its tensile strength was approximately 50% lower than for electrospun fibrinogen, which might be due to the fact that these scaffolds were prepared with much higher protein concentrations and treated with different cross-linking agents.^20,22,23^

The elongation at break decreased from 31% at 30 min to 13% at 240 min cross-linking time (see Figure 2C), a range similar to previous findings.^27^ Overall, elongation at break decreased with longer cross-linking time, which opposed the trend of the Young’s modulus, as expected for stiffer samples. Non-cross-linked fibrin references showed a significantly higher elongation at break of 59%, notably lower than previous results of 170%.^27^ Overall, the elongation at break of fibrinogen scaffolds with varied cross-linking time decreased by an order of magnitude compared to electrospun fibrinogen (100–230%).^20,22^ This result supports the hypothesis that self-assembled fibrinogen is less ductile due to its higher compactness.^27^

No clear trend in toughness with varying cross-linking time was observed (see Figure 2D). Median toughness values for cross-linking times of 30, 60, 120, and 240 min were 16.2 kJ/m^3^, 13.8 kJ/m^3^, 17.1 kJ/m^3^, and 10.8 kJ/m^3^, respectively. These values were slightly below our previous reports for self-assembled fibrinogen.^27^ Non-cross-linked fibrin references yielded the highest toughness at 57.6 kJ/m^3^, consistent with our earlier findings,^27^ albeit with the highest standard deviation. Since no significant differences were found for different cross-linking times, we conclude that it did not affect the scaffold toughness.

The drapability of skin substitutes is essential to permit motion during wound healing and to avoid mechanical tension in the wound.^39^ We found a mean bending stiffness of 0.19 ± 0.10 mg·cm (*n* = 16) and 0.25 ± 0.19 mg·cm (*n* = 17) for fibrinogen nanofibers cross-linked for 60 and 120 min, respectively. Scaffolds cross-linked for 60 min were harder to remove from the PDMS mold than those cross-linked for 120 min, which is consistent with the increasing trend in bending stiffness despite the high standard deviations observed. Interestingly, the bending stiffness of self-assembled fibrinogen was lower than that of electrospun nylon scaffolds, for which a bending stiffness of 1.4 to 2.9 mg·cm was reported.^40^ This difference presumably originates from the larger diameter of nylon fibers between 250 and 650 μm^40^ compared to the 100 to 300 nm self-assembled fibrinogen nanofibers used in this study. Despite the observed standard deviations, our simple setup can provide a good basis to measure the bending behavior of various biopolymer scaffolds, like collagen nanofibers.^41^ To further advance our concept, a comparison with established methods for the analysis of fiber composite preforms, preimpregnated fibers,^42^ and macroalgae^43^ will be crucial.

For electrospun scaffolds from various (bio)polymers, immersion in aqueous media during cell culture changes the mechanical properties and reduces porosity, which can hinder cell infiltration during skin repair.^4,5,44^ On the other hand, adequate swelling is needed to absorb wound exudates and hydrate the wound.^7,45^ Thus, we studied how cross-linking times affect scaffold swelling by measuring thickness and mass changes upon rehydration using 15 mg fibrinogen bulk scaffolds. The caliper method yielded an average thickness of 41 μm for 60 min and 40 μm for 120 min cross-linking time, while 47 and 41 μm were found by optical analysis, respectively (see Supporting Information, Table S3). No significant differences were found between both measurement methods, with the caliper method being faster but more error-prone due to variations in the closing pressure. Interestingly, both methods and cross-linking times showed a lower thickness for rectangular samples than dog-bone shaped scaffolds (compare Table S2 and Table S3), probably due to different drying times for the two sample geometries.

Subsequently, we measured the mass of 60 and 120 min cross-linked bulk scaffolds with 15 mg fibrinogen after incubation at varying humidity (see Table 1). For 60 min, we obtained a dry mass of 33 mg at 0% humidity and 60 °C, which increased by 8.8% at 65% humidity and 20 °C and by 22% at 85% humidity and 20 °C in comparison to dry scaffolds. The mass of 120 min cross-linked scaffolds was 42 mg in the dry state and increased by 8.0% at 65% humidity and by 22% at 85% humidity. Interestingly, the scaffold mass exceeded the bare protein amount by 100% or more, which can be attributed to residual salt in the scaffolds. At all humidity levels, 120 min cross-linked fibrinogen scaffolds had a mass approximately 27% higher than for 60 min cross-linked scaffolds. Hence, we assume that scaffolds prepared with shorter cross-linking time may contain more non-cross-linked proteins that dissolved upon scaffold washing. In addition, varying degrees of Na^+^ ions from the PBS solution remaining in the cross-linked scaffolds after washing and drying^26^ will most likely contribute to their mass. Overall, humidity primarily affected water uptake in fibrinogen scaffolds, while cross-linking time had no effect. This may allow for adjustment of mechanical scaffold properties while maintaining wound hydration during tissue regeneration.^45^

**Table 1:** Mass of Bulk Scaffolds with 15 mg Fibrinogen in Dependence on the Cross-Linking Time, and Relative Humidity and Temperature during Incubation in a Climate Chamber^a^

Cross-linking time	Mass while dry (60 °C)	Mass at 65% air humidity and 20 °C	Mass increase	Mass at 85% air humidity and 20 °C	Mass increase in percent
60 min	33.07 mg	36.00 mg	8.8%	40.50 mg	22.4%
120 min	42.10 mg	45.47 mg	8.0%	51.50 mg	22.0%

a For both cross-linking times, *n* = 3 scaffolds were analyzed. The relative mass increase is given in percent with regard to the dry mass for the respective cross-linking time.

### 3.3 Effect of Scaffold Layering on the Mechanical Scaffold Properties

To fabricate three-dimensional (3D) scaffolds for the treatment of large defects in soft tissue like cartilage or skin,^2,46^ the thickness of nanofibrous fibrinogen scaffolds needs to be increased. To achieve a scaffold upscaling through layering, we introduced successive self-assembly and first measured the scaffold thickness in the dry state (see Supporting Information, Table S4). For bulk and six-layer scaffolds assembled with 15 mg protein, the average thickness ranged from 57 to 67 μm, doubling to 102 to 110 μm with 30 mg protein. Three-layer scaffolds with 30 mg fibrinogen had the lowest thickness of 41 μm, close to controls with 15 mg fibrin prepared with one layer (46 μm) or three layers (51 μm). These results show that scaffold thickness directly correlates with the protein amount, independent of the layer structure.

**Figure 3:**
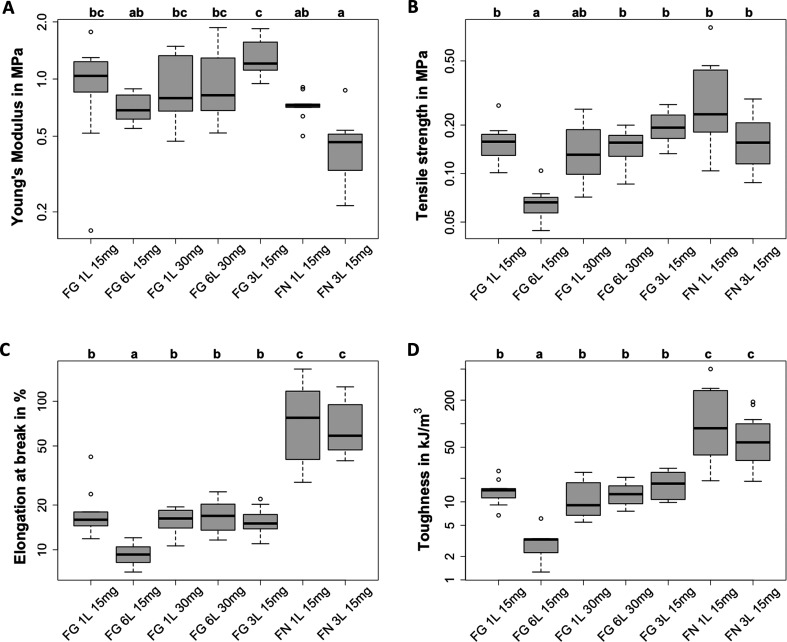
**Effect of scaffold layering on the mechanics.** Mechanical characteristics of fibrinogen (FG) and fibrin (FN) scaffolds with protein amounts of 15 or 30 mg deposited in one layer (1L), three layers (3L), or six layers (6L) presented on a logarithmic *y*-axis: (A) Young’s modulus, (B) tensile strength, (C) elongation at break and (D) toughness. The sample size varied from *n* = 7 (FG 6L 15 mg), *n* = 8 (FG bulk 30 mg and FG 6L 30 mg), *n* = 9 (FN bulk 15 mg and FG 3L 15 mg), *n* = 10 (FG bulk 15 mg) to *n* = 12 (FN 3L 15 mg). All scaffolds were cross-linked in FA vapor for 120 min. Significant differences between sample parameters are indicated by different letters above the boxplot. Samples sharing at least one letter do not differ significantly from each other.

Following rehydration in demineralized water, we found a median Young’s modulus between 0.71 and 1.00 MPa for both 15 mg and 30 mg fibrinogen scaffolds in single- and six-layer design (see Figure 3A). The highest Young’s modulus of 1.32 MPa was obtained for three-layer scaffolds with 15 mg protein, consistent with native fibrin^47^ and our previous study.^27^ Compared to the Young’s modulus of electrospun fibrinogen^20,22^ and elastin scaffolds^48^ (0.2 to 0.6 MPa and 0.1 to 0.2 MPa, respectively), self-assembled fibrinogen exhibited a higher stiffness regardless of the layer number or protein amount and are therefore less ductile. Bulk fibrin samples had a median Young’s modulus of 0.73 MPa, while three-layered fibrin showed the lowest value of 0.45 MPa, indicating more elastic behavior than nanofibrous fibrinogen, probably due to thrombin-mediated cross-linking. Interestingly, these values surpass the Young’s modulus of fibrin gels by 1 order of magnitude.^49^ However, these gels were prepared with aprotinin and thrombin using NaCl instead of PBS,^49^ which altered the cross-linking state and thus possibly influenced fibrin mechanics.^50^ Despite statistical variations, no clear trend was found linking the Young’s modulus to layer number or protein amount. Overall, the Young’s modulus values of all fibrinogen scaffolds align well with the mechanics of native skin, for which values between 0.01 and 50 MPa were previously reported.^7^

The tensile strength showed consistent trends across most layering parameters and fibrinogen amounts, ranging from 0.13 to 0.19 MPa (see Figure 3B). Six-layer fibrin with 15 mg fibrinogen exhibited a comparable tensile strength of 0.16 MPa, slightly differing for bulk design at 0.23 MPa. These values agree well with our previous findings for three-layer fibrinogen and fibrin with 15 mg protein (0.2 MPa^27^) and are also in the same range as for electrospun fibrinogen (0.3 to 0.5 MPa^20,22^ and 0.2 MPa^48^). Notably, six-layer scaffolds with 15 mg fibrinogen showed a significantly lower median tensile strength of 0.07 MPa, resembling more ductile fibrin matrices (0.01 to 0.03 MPa^51^). This suggests reduced cohesion between individual fibrinogen layers in six-layer scaffolds for low protein amounts of 15 mg.

Layered fibrinogen scaffolds exhibited a similar trend in elongation at break as tensile strength (see Figure 3C). The significantly lowest median value of 9% was found for six layers with 15 mg protein, likely due to low interlayer cohesion. Elongation at break ranged from 15% to 17% for all other scaffolds, consistent with our previous findings,^27^ and no dependence of layer number or protein amount was found. However, compared to electrospun fibrinogen (elongation between 100% and 230%^20,22^), self-assembled fibrinogen showed reduced elongation at break by a factor of 6 to 14. For bulk and three layers, respectively, 15 mg fibrin yielded significantly higher elongation at break values of 59% and 77%, that were below the range of fibrin prepared with varying pH and fibrinogen concentrations (extending by 100% to 200%).^52^ Overall, these data highlight the lower ductility of self-assembled fibrinogen nanofibers, independent of layer number or protein amount.

The lowest median toughness of 3.3 kJ/m^3^ was observed for six-layer scaffolds with 15 mg fibrinogen (see Figure 3D), while other samples ranged from 9.0 to 17.1 kJ/m^3^. Notably, these values, particularly for bulk scaffolds, are below the previously reported range of 22.2 to 24.0 kJ/m^3^.^27^ Overall, toughness also showed no clear dependence on scaffold layering or protein amount. Both fibrin scaffolds exhibited significantly higher toughness values between 57.6 and 87.5 kJ/m^3^, albeit lower than the previously reported 169.8 kJ/m^3^.^27^ These slight variations in toughness may stem from study-related differences, such as variations in fibrinogen batches used. However, no other studies have reported on the toughness of nanofibrous fibrinogen or fibrin hydrogels, limiting further comparisons.

In summary, scaffold layering did not significantly influence the mechanical properties of nanofibrous fibrinogen scaffolds. Since different fibrinogen scaffolds were associated with varying concentrations of fibrinogen (see Table S1), we conclude that this parameter did not influence the scaffold mechanics, which is in contrast to previous reports for electrospun fibrinogen nanofibers.^22^ For future applications in wound healing, scaffolds in bulk design will be best suited as they are easier to assemble and require only one incubation step, reducing the fabrication time and improving batch-to-batch consistency.

### 3.4 Influence of Freeze-Drying on the Pore Size and Mechanical Characteristics

**Figure 4:**
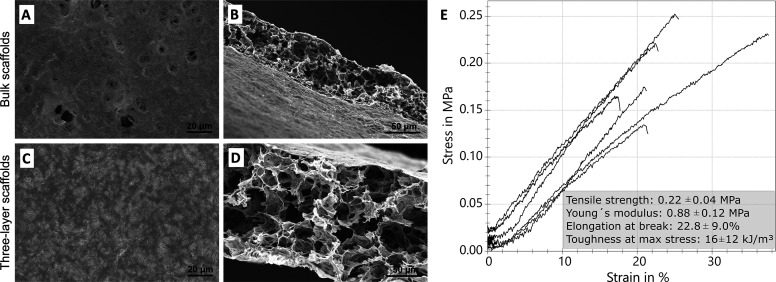
**Influence of freeze-drying on scaffold morphology and mechanics.** (A–D): SEM images of freeze-dried fibrinogen scaffolds assembled with 15 mg protein and different layer numbers. (A) Bulk scaffold exhibits a coherent surface layer with (B) a porous core that appeared more compact than in (C) three-layer scaffolds, which showed a coherent surface layer with more fibrous features, and (D) a highly porous core. (E) Stress–strain curves and tensile characteristics (median values ± mean arithmetic deviation (MAD)) of seven freeze-dried fibrinogen scaffolds assembled with bulk design and 15 mg protein.

Scaffold porosity plays a crucial role during wound healing^7^ since an interconnected pore structure together with sufficient mechanical integrity is required to enable biophysical and -chemical signaling between different cells.^4^ Here, we used freeze-drying, a method established for cryopreservation of skin allografts^4^ and performed cross-sectional SEM analysis of three-layer and bulk scaffolds with 15 mg fibrinogen. As to be expected for this technique,^53^ freeze-drying preserved the porous scaffold architecture. For both scaffolds, we found rather flat surfaces with more fibrous features in three-layer samples (see Figure 4A and 4C). Interestingly, the coherent top layer differed from the undulated fiber topography we had found after air-drying of fibrinogen scaffolds at room temperature.^24,25,27^ Surface-dependent inhomogeneities during freeze-drying may account for this difference.^53^ Previously, air-drying led to collapsed pores with a puff pastry-like structure,^24^ whereas freeze-drying now yielded an interconnected pore network below the coherent surface (see Figure 4B and D). Thus, porous fibrinogen resembled freeze-dried collagen scaffolds, albeit with larger pores,^54,55^ and was also similar to the structure of a bird’s skull,^56^ consisting of hard outer layers and foam-like inner bone structure or an engineered polymer foam sandwich structure.^57^

For freeze-dried fibrinogen, we found average pore sizes of 20 ± 9 μm in comparison to only 7 μm for electrospun fibrinogen.^19,22^ The obtained value serves as a good approximation for the effective pore size that characterizes which cells can migrate into a tissue engineering scaffold. For a more detailed analysis of the effective pore size, alternative techniques could be used in the future, such as mercury porosimetry and microcomputed tomography^58^ or environmental scanning electron microscopy, which would facilitate analysis of hydrated scaffolds.^59^ Human skin cells are typically 20 to 40 μm in size,^10^ which limits cell infiltration into electrospun scaffolds.^60^ Hence, time-consuming postprocessing is required to enlarge the pores, as it was used for electrospun elastin and gelatin for skin cell cultures.^61^ Toward wound healing applications, the porous architecture of freeze-dried fibrinogen nanofibers could support nutrient supply and gas exchange.^7^ Freeze-drying also increases the shelf life and storage durability of fibrinogen nanofibers, which is crucial for skin substitutes,^13,62,63^ in addition to simple preparation without pore expansion.

Tensile testing of freeze-dried scaffolds yielded a Young′s modulus of 0.88 ± 0.12 MPa, a tensile strength of 0.22 ± 0.04 MPa, an elongation at break of 22.8 ± 9.0%, and a toughness of 16 ± 12 kJ/m^3^ (see Figure 4E). Thus, freeze-dried bulk samples showed similar mechanical properties to air-dried and rehydrated scaffolds with only minor deviations (c.f. Figure 3), indicating that the overall mechanical properties were maintained during freeze-drying. In comparison, freeze-dried scaffolds were slightly less rigid, but had higher strength, elongation and toughness than air-dried scaffolds. These characteristics will be advantageous for wound healing materials that need to offer increased resistance to tearing and enable greater flexibility in delicate wound environments, which often feature uneven surfaces.^13^

In general, the mechanical properties of skin are highly anisotropic and depend on the body site, age, and gender^9,11^ as well as the measurement method and sample orientation with regard to the Langer lines.^12^ These diverse factors make it very difficult to compare the mechanical properties of skin across different studies. Interestingly, most studies report the Young’s modulus of the skin as its main mechanical characteristic while other properties like fracture toughness, tensile strength and elongation at break are usually not considered.^9,11,64^ Published values for the Young’s modulus of different skin samples obtained by tensile testing, indentation or suction tests range from only 5 kPa to 140 MPa.^11,12^ Considering this extremely wide range, the Young’s modulus of 0.54 to 1.34 MPa we measured by tensile testing of our air- or freeze-dried fibrinogen scaffold types falls well into this range. Since skin substitutes and scaffolds for wound healing need to mimic the mechanical properties of native skin^2,7,63^ we conclude that self-assembled fibrinogen nanofibers offer good potential for this application.

### 3.5 Time-Dependent Degradation of Fibrinogen Scaffolds

**Figure 5:**
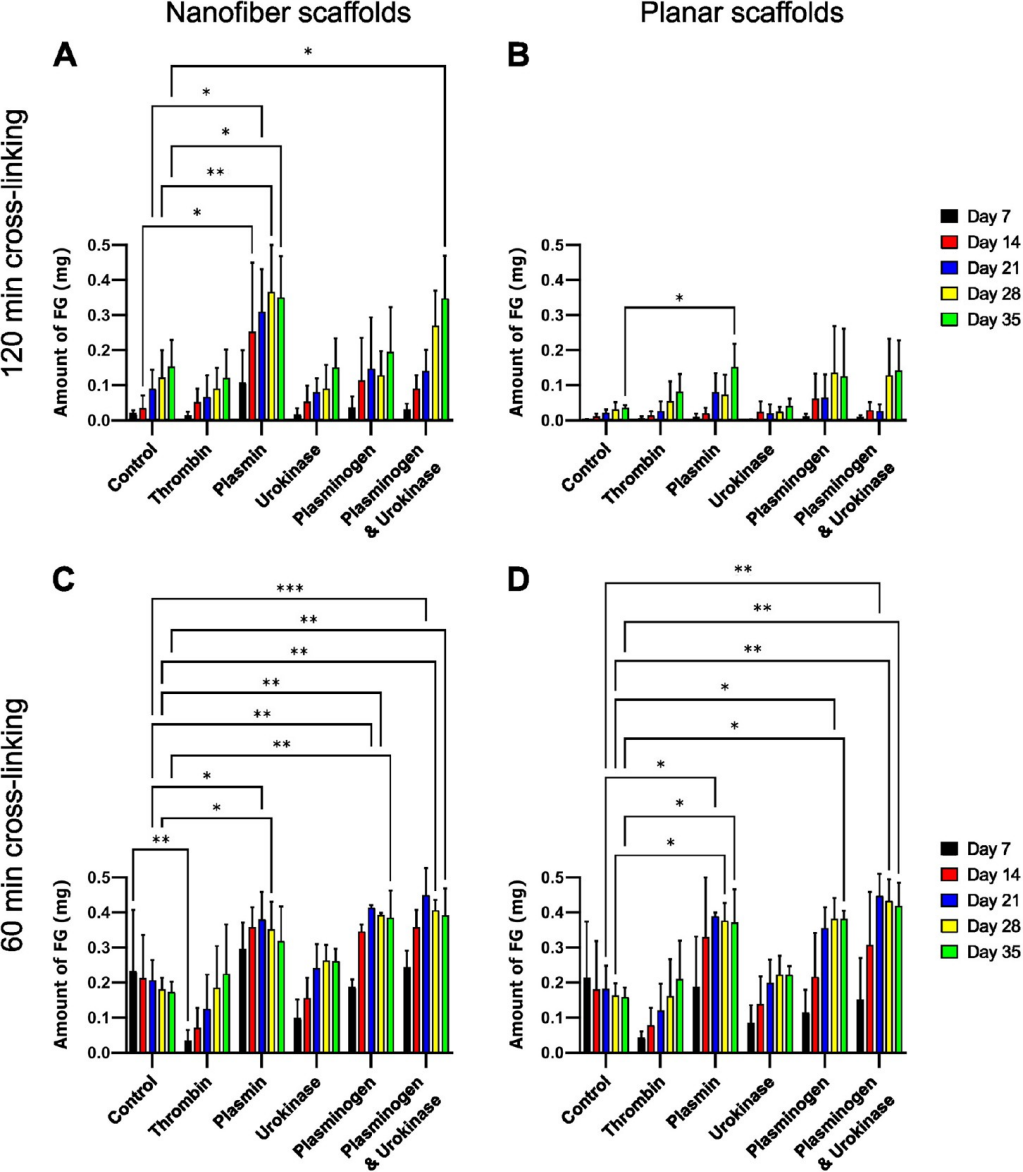
**Long-term enzymatic degradation of nanofibrous and planar fibrinogen scaffolds.** The amount of fibrinogen released into the supernatant after 35 days of incubation in the absence (control) or presence of the respective enzymes in HEPES buffer of fibrinogen nanofiber scaffolds (A, C) or planar fibrinogen scaffolds (B, D), which were cross-linked with FA vapor for 120 min (A, B) or 60 min (C, D), was calculated using absorbances measured at 280 nm. A time-dependent increase in fibrinogen release was observed for all scaffold types, with plasmin and a combination of plasminogen and urokinase showing the highest amounts. 60 min cross-linked scaffolds of both topographies showed more fibrinogen release, indicating a higher susceptibility to degradation than 120 min cross-linked scaffolds.

Cross-linking was previously found to influence degradation of electrospun fibrinogen scaffolds.^19,20,22^ As one of the first steps during blood coagulation, thrombin cleaves off fibrinopeptides A and B, thus converting fibrinogen to fibrin.^65^ Urokinase activates plasminogen and converts it to plasmin, which directly binds to fibrin to induce fibrinolysis.^66^ Therefore, we studied the effect of these enzymes on the degradation of fibrous and planar fibrinogen scaffolds cross-linked with FA vapor for either 60 or 120 min. Over 35 days, we observed varying fibrinogen release into the supernatant compared to enzyme-free controls (see Figure 5). Nanofibers cross-linked for 120 min exhibited a time-dependent increase in fibrinogen release for all enzyme treatments (see Figure 5A), analogous to the degradation of fibrin scaffolds described earlier.^67^ A significant effect was found with plasmin treatment, except at day 7, and a combination of plasminogen and urokinase increased fibrinogen release significantly after 35 days. No significant differences were found for thrombin, urokinase, and plasminogen treatment of 120 min cross-linked nanofibers. On the other hand, planar scaffolds cross-linked for 120 min showed minimal fibrinogen release over time for all enzymes, except for a significant increase with plasmin treatment on day 35 (see Figure 5B).

Comparatively, 60 min cross-linked scaffolds yielded higher fibrinogen release for both topographies over 35 days that often already reached a saturation after 21 days (see Figure 5C, D). These data show that a lower cross-linking time correlated with a higher susceptibility to degradation. Interestingly, higher amounts of fibrinogen were detected in the control of nanofibrous and planar fibrinogen without enzymes, indicating some enzyme-independent degradation for 60 min cross-linked scaffolds only in the presence of HEPES buffer. Overall, a time-dependent increase of fibrinogen release was observed for both fibrinogen topographies with all enzyme treatments (see Figure 5C, D). Significant increases were found for nanofibrous and planar fibrinogen scaffolds incubated with plasmin, plasminogen and plasminogen/urokinase combination for 21, 28, and 35 days. Thrombin and urokinase showed no significant differences. In summary, our results show that 60 min cross-linked scaffolds were more susceptible to time-dependent degradation accelerated by certain enzymes, which is consistent with a previous study on trypsin-based degradation of macroporous gelatin–fibrinogen scaffolds.^68^ Overall, nanofibrous scaffolds were found to release more fibrinogen than planar scaffolds, which may be attributed to their 15-fold higher surface roughness.^27^

**Figure 6:**
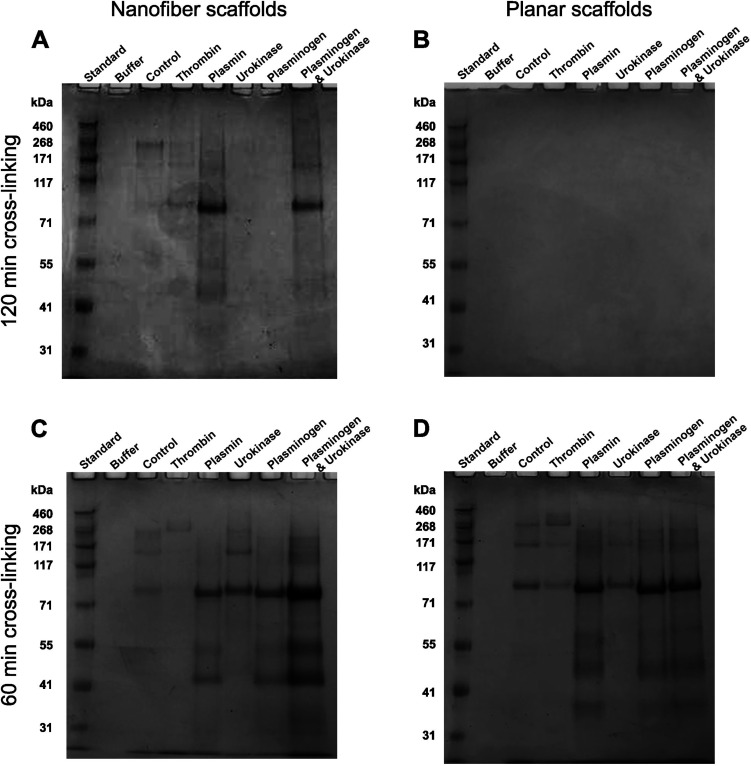
**Molecular size of degradation products after 35 days of enzymatic digestion**. The products present in the supernatant of enzymatically digested fibrinogen nanofiber scaffolds (A, C) or planar fibrinogen scaffolds (B, D), which were cross-linked with FA vapor for 120 min (A, B) or 60 min (C, D), were analyzed via SDS-PAGE after 35 days of incubation in the absence (control) or presence of respective enzymes in HEPES buffer. HEPES buffer control was additionally analyzed as a negative control (Buffer). The molecular sizes of the degraded products were compared against a protein standard that was run on each gel. 60 min cross-linked scaffolds showed comparatively more protein fragment bands than 120 min cross-linked scaffolds, indicating a higher degree of scaffold degradation. Most prominent bands were observed when scaffolds were treated with plasmin and a combination of plasminogen and urokinase.

SDS-PAGE analysis of degraded protein products after 35 days showed no effect on nanofibrous or planar fibrinogen scaffolds in the presence of HEPES alone, as indicated by the absence of bands on all SDS-PAGE gels (see Figure 6A to D, column “Buffer”). When 120 min cross-linked nanofiber scaffolds were treated with plasmin or a plasminogen/urokinase combination, prominent bands appeared between 117 kDa and 71 kDa. In addition, we found smears from 268 kDa to 117 kDa for the control, thrombin, plasmin, and plasminogen/urokinase treatments, with further fragments detected between 55 kDa and 41 kDa for the latter two (Figure 6A). No bands were visible when 120 min cross-linked fibers were treated with urokinase or plasminogen alone. 120 min cross-linked planar scaffolds treated with all enzymes showed no protein bands, indicating that no digested fragments were present (see Figure 6B).

When 60 min cross-linked nanofiber and planar scaffolds were incubated in enzyme-free HEPES buffer for 35 days, fragments ranging from 268 kDa to 117 kDa and a fairly prominent band between 117 kDa and 71 kDa were found (see Figure 6C, D), indicating some enzyme-independent degradation, consistent with previous fibrinogen release analysis (see Figure 5). Various protein bands were detected for enzyme treatment of both scaffold types (see Figure 6C, D). Protein fragments between 268 kDa and 117 kDa were found with thrombin, urokinase, and a plasminogen/urokinase combination for both fibrinogen topographies. Faint bands between 117 kDa and 72 kDa were seen with thrombin and urokinase, becoming more pronounced with plasmin, plasminogen, and plasminogen/urokinase (see Figure 6C, D). Additionally, smaller fragments between 55 kDa and 31 kDa were visible with plasmin, plasminogen, and plasminogen/urokinase. The prominent protein fragment bands between 117 kDa and 71 kDa observed here were consistent with short-term plasmin digestion of soluble fibrinogen (see Supporting Information, Figure S2), lacking the native undigested fibrinogen band at around 340 kDa, as anticipated for enzymatic scaffold treatment.

Overall, 60 min cross-linked fibrinogen scaffolds exhibited more protein fragments than 120 min cross-linked ones, indicating greater degradation with shorter cross-linking time and thus confirming our protein release analysis (see Figure 5) and previous studies.^67,68^ For 60 min cross-linking, this trend was consistent for both fibrinogen topographies, whereas more degradation products were observed in nanofibers than in planar scaffolds for 120 min cross-linking, again indicating an effect of the increased accessible surface area.^27^

**Figure 7:**
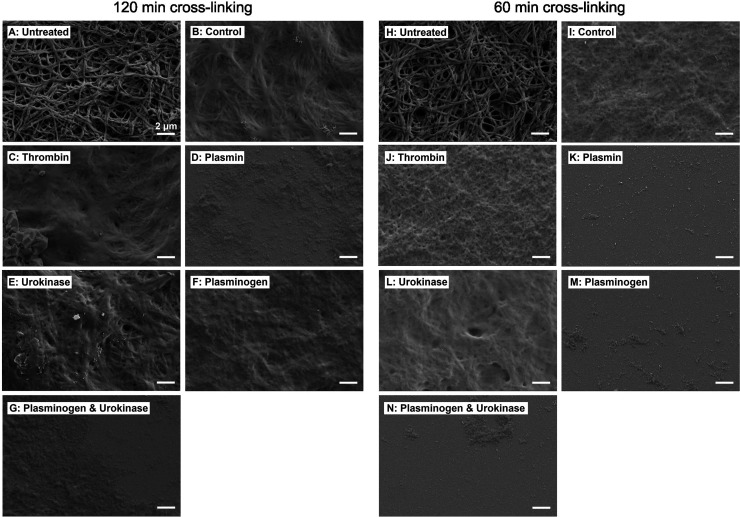
**SEM images of degraded fibrinogen nanofiber scaffolds.** Fibrinogen nanofiber scaffolds were cross-linked with FA vapor for 120 min (A–G) or 60 min (H–N) and were dried without prior incubation (untreated) or were incubated for 35 days in a HEPES buffer (control) or in a HEPES buffer containing respective enzymes, before being subjected to SEM imaging. Fiber structures could be observed, although they had merged into an almost confluent top layer after 35 days of incubation in comparison to untreated fibers, whereas a treatment with plasmin or a combination of plasminogen and urokinase completely degraded the fiber morphology.

Topographical SEM analysis after 35 days of enzyme treatment revealed significant variations for fibrinogen nanofibers (see Figure 7). SEM images were compared to nanofibers cross-linked with FA vapor for 120 min (see Figure 7A) or 60 min (see Figure 7H) that exhibited distinct layers of dense and porous nanofibers. Both controls incubated in HEPES buffer revealed a less defined fiber topography without pronounced pores irrespective of the cross-linking time (see Figure 7B, I), possibly due to prolonged exposure to an aqueous environment. This observation differed from our previous studies, in which the porous topography of 120 min cross-linked fibrinogen nanofibers incubated in DMEM cell culture medium for up to 2 weeks did not change.^27−29^ The long-term analysis of nanofibers in HEPES alone (see Figure 5A) also shows that fibrinogen release increased mainly after 21 days, which presumably led to the observed changes in fiber topography.

Similar merged fibers, that had fused into a confluent top layer, were observed with thrombin, urokinase, or plasminogen treatment for 120 min cross-linked fiber scaffolds (see Figure 7C, E, F), and with thrombin and urokinase treatment for 60 min cross-linked fibers (see Figure 7J, L), which aligns well with the low fibrinogen release we found for these settings (see Figure 5A and 5C). The distinct nanofibrous topography almost completely disappeared in 120 min cross-linked scaffolds treated with plasmin and a plasminogen/urokinase combination (see Figure 7D, G), leaving only sparse fibrous remnants. Similarly, 60 min cross-linked scaffolds treated with plasmin, plasminogen, and a plasminogen/urokinase combination (see Figure 7K, M, N) exhibited a smooth surface similar to planar scaffold topography (see Supporting Information, Figure S3A, H), indicating loss of nanofibrous features, which agrees well with the previously found trends in fibrinogen release (see Figure 5) and degradation products (see Figure 6). In contrast, although a release of fibrinogen from planar scaffolds was found in dependence of the cross-linking time (see Figure 5 and Figure 6D), the smooth topography remained unchanged after 35 days in HEPES buffer or exposure to any of the enzymes compared to untreated planar scaffolds (see Supporting Information, Figure S3). This means that topographical changes driven by enzymatic degradation are a parameter that only needs to be considered for fibrinogen nanofibers when designing new scaffold materials for regenerative medicine.

All of the above methods revealed a pronounced degradation of fibrinogen scaffolds in the presence of plasmin and plasminogen/urokinase combination that depended on the cross-linking time and fibrinogen topography. Surprisingly, thrombin, the only enzyme in our study that binds directly to fibrinogen,^65^ resulted in only minor fibrinogen degradation and topographical changes comparable to those of HEPES buffer alone. Hence, we assume that thrombin could not bind to fibrinogen in the nanofibrous or planar scaffolds since they had undergone cross-linking in FA vapor. This treatment involves a reaction of aldehyde groups with lysine residues in the protein molecule^69^ that might have made it impossible to cleave off the fibrinopeptides. Although plasmin binds directly to fibrin *in vivo* by activation of plasminogen by urokinase^66^ and the degradation of fibrin hydrogels has also been observed under *in vitro* conditions,^70^ we found a strong degradative effect of this enzyme on fibrinogen scaffolds in all our experiments. A similar level of fibrinogen degradation was found for a combination of urokinase and plasminogen. Since urokinase alone and plasminogen alone did not degrade fibrinogen scaffolds to the same extent, this observation may indicate that urokinase was able to activate plasminogen and convert it to plasmin in our experimental setup. Interestingly, for both, planar and fibrinogen nanofibers that were cross-linked for 60 min, plasminogen alone yielded a degradation close to that of plasmin and plasminogen/urokinase combination as indicated by a significant fibrinogen release toward the end of the incubation (see Figure 5C and D) and degradation products in SDS-PAGE gels after 35 days (see Figure 6C and D). One possible explanation for plasminogen activation without urokinase may be that plasminogen alone is able to directly bind to fibrinogen^71^ to induce fibrinogenolysis.^72^ We assume that plasminogen-induced degradation could only occur for 60 min cross-linking time because these scaffolds exhibited a lower cross-linker density compared to 120 min cross-linked scaffolds, which is associated with higher diffusion rates for water and small molecules.^73^

To tailor the stability of self-assembled fibrinogen scaffolds for selected applications in soft tissue engineering, it will be important also to study the degradative effect of different matrix metalloproteinases.^70,74,75^ If lower degradation rates are required, mixing fibrinogen with synthetic polymers such as polyethylene glycol,^76^ poly(l-lactic acid)-*co*-poly(epsilon-caprolactone),^77^ or polycaprolactone^78^ could also be a promising approach to be explored for the preparation of fibrinogen-based scaffolds by salt-induced self-assembly.

## 4 Conclusion

In conclusion, optimal resistance to mechanical damage and enzymatic degradation of self-assembled fibrinogen nanofibers upon rehydration was achieved by cross-linking with formaldehyde vapor, whereas treatment with liquid aldehydes, genipin, EDC, and transglutaminase did not preserve the nanofibrous architecture. We found that layering did not significantly influence the mechanical properties but affected the architecture and thickness of nanofibrous fibrinogen scaffolds. Cross-sectional analysis showed average pore sizes of 20 ± 9 μm, which will be beneficial to support cell infiltration and nutrient supply. Freeze-drying preserved the mechanical properties and porous architecture, crucial for good storage stability and increased shelf life. Increased cross-linking times enhanced scaffold stiffness and decreased the elongation while scaffold swelling remained unaffected. At the same time, longer cross-linking times were found to reduce scaffold degradation by various enzymes with plasmin and a combination of urokinase and plasminogen showing the strongest degradative effect. Due to their high specific surface area, nanofibrous scaffolds were more susceptible to enzymatic degradation than planar scaffolds. Overall, our findings provide a good basis to develop self-assembled fibrinogen fiber scaffolds with tailored structure–function relationships and degradation behavior for skin and soft tissue engineering.

## Supporting Information Available

Further information on the methods, additional data on scaffold thickness and degradation of soluble fibrinogen, SEM images of planar fibrinogen after enzymatic degradation (PDF)
