# Supplementary material for: Structure, Properties and Degradation of Self-Assembled Fibrinogen Nanofiber Scaffolds
Source: ACS Appl Bio Mater. 2024 Sep 3;7(9):6186–200. doi: 10.1021/acsabm.4c00761 (PMC11409215; doi:10.1021/acsabm.4c00761)
Supplement: Supplementary file 1 — mt4c00761_si_001.pdf [file mt4c00761_si_001.pdf]

Supporting information

Structure, properties and degradation of self-assembled fibrinogen nanofiber scaffolds

Till Strunk[1,§], Arundhati Joshi[1], Mahta Moeinkhah[1], Timon Renzelmann[1], Lea Dierker[2], Dietmar Grotheer[3], Nina Graupner[4], Jörg Müssig[4], Dorothea Brüggemann[1,5,#,*]

[1] Institute for Biophysics, University of Bremen, Otto-Hahn-Allee 1, 28359 Bremen, Germany

[2] Hochschule Bremen - City University of Applied Sciences, Neustadtswall 30, 28199 Bremen, Germany

[3] Chemical Process Engineering, Faculty of Production Engineering, University of Bremen, Leobener Str. 6, 28359 Bremen, Germany

[4] HSB – City University of Applied Sciences, Dept. of Biomimetics, The Biological Materials Group, Neustadtswall 30, 28199 Bremen, Germany

[5] MAPEX Center for Materials and Processes, University of Bremen, 28359 Bremen, Germany

[§] Present address: Department of Biomedical Engineering, Medical Additive Manufacturing Research Group (Swiss MAM), University of Basel, Hegenheimermattweg 167C, 4123 Allschwil, Switzerland

[#] Present address: Biophysics and Applied Biomaterials, Hochschule Bremen - City University of Applied Sciences, Neustadtswall 30, 28199 Bremen, Germany

[*] Corresponding author: dorothea.brueggemann@hs-bremen.de

S1: Preparation of nanofibrous fibrinogen scaffolds for mechanical testing

***Table S1**: Preparation of self-assembled fibrinogen (FG) nanofibers with varying amounts of layers and different protein amounts, yielding a final Fg:PBS ratio of 2:1 in all samples. To prepare fibrin (FN) scaffolds, different thrombin concentrations in PBS were added to fibrinogen.*

Sample	FG volume	PBS	Thrombin in PBS
FG bulk 15 mg	1.25 ml of 12 mg/ml: once	0.75 ml of 20x PBS: once	-
FG 6L 15 mg	1 ml of 2.5 mg/ml: 6 times	1 ml of 2.5x PBS: 6 times	-
FG bulk 30 mg	2.5 ml of 12 mg/ml: once	1 ml of 30x PBS: once	-
FG 6L 30 mg	1 ml of 5 mg/ml: 6 times	1 ml of 5x PBS: 6 times	-
FG 3L 15 mg	1 ml of 5 mg/ml 3 times	1 ml of 5x PBS: 3 times	-
FN bulk 15 mg	1.25 ml of 12 mg/ml once	-	0.75 ml of 40 U/ml in 20x PBS: once
FN 3L 15 mg	1 ml of 5 mg/ml 3 times	-	1 ml of 10 U/ml in 5x PBS: 3 times

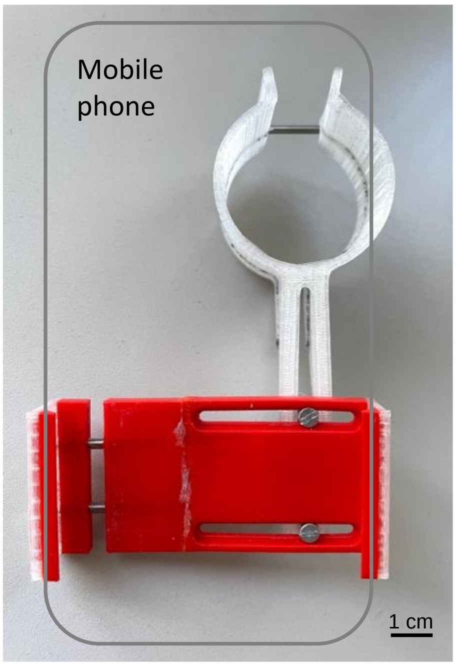

***Figure S1: Adjustable 3D-printed mobile phone holder.** The holder was attached to the microscope eyepiece for thickness measurements of fibrinogen scaffolds.*

Table S2: *Thickness and cross-sectional area of wet fibrinogen (FG) and fibrin (FN) scaffolds with a dog-bone-shape prepared with varying cross-linking times and 15 mg protein were measured with a caliper after tensile testing and averaged for each sample type. All scaffolds had a three-layer design and an overall width of 8 mm.*

**Sample Type**	**Average thickness (± std. dev.) in µm**	**Cross-sectional area in mm^2**
FG 30 min	45 ± 5	0.360 ± 0.040
FG 60 min	48 ± 1	0.384 ± 0.008
FG 120 min	41 ± 2	0.328 ± 0.016
FG 240 min	46 ± 4	0.368 ± 0.032
FN 3L 15 mg	52 ± 1	0.416 ± 0.008

Table S3: *Thickness measurements of rehydrated bulk scaffolds containing 15 mg fibrinogen that were cross-linked for 60 min or 120 min. Rectangular scaffolds were 16 mm x 32 mm in size and measured either with a caliper or under an optical microscope.*

**Measurement method**	**Cross-linking time in min**	**Average thickness ± SD in µm**
Caliper	60 (n = 16)	41 ± 6
Caliper	120 (n = 13)	40 ± 9
Optical	60 (n = 16)	47 ± 13
Optical	120 (n = 17)	41 ± 7

Table S4: *Thickness and cross-sectional area of wet fibrinogen (FG) and fibrin (FN) scaffolds with dog-bone-shape prepared with varying layer number and protein amount were measured with a caliper after tensile testing and averaged for each sample type. All scaffolds were cross-linked for 120 min and had a width of 8 mm.*

**Samples Type**	**Average thickness (± std. dev) in µm**	**Cross-sectional area in mm^2**
FG 1L 15 mg	68 ± 17	0.544 ± 0.136
FG 6L 15 mg	57 ± 8	0.456 ± 0.064
FG 1L 30 mg	110 ± 30	0.880 ± 0.240
FG 6L 30 mg	102 ± 6	0.816 ± 0.048
FG 3L 15 mg	41 ± 2	0.328 ± 0.016
FN 1L 15 mg	46 ± 3	0.368 ± 0.024
FN 3L 15 mg	52 ± 1	0.416 ± 0.008

S2: Molecular size of products of serine protease-digested soluble fibrinogen

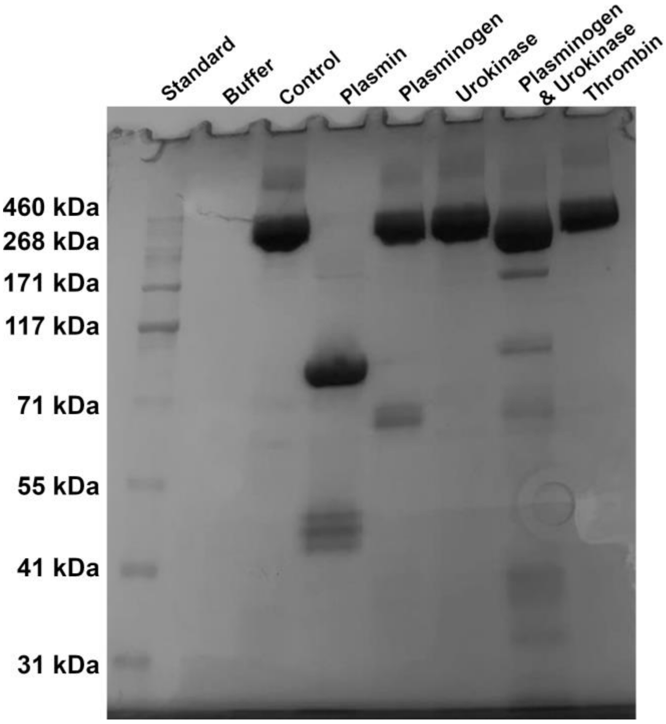

Figure S2: Molecular size of degradation products after short-term digestion of fibrinogen solution. *As a reference for long-term enzymatic fibrinogen scaffold degradation, the degradation of fibrinogen in solution was analyzed to evaluate the size of the degraded products. For this, 1 mg/ml fibrinogen in HEPES buffered saline was incubated at 37 °C overnight in the absence or presence of the respective enzymes and subsequently analyzed via SDS-PAGE. HEPES buffer control was additionally analyzed as a negative control (Buffer). The molecular sizes of degraded products were compared against a protein standard. A prominent band between 460 kDa and 268 kDa, indicative of native fibrinogen protein having a molecular size of 340 kDa, was observed for control, plasminogen, urokinase, and thrombin treatments, whereas this band was slightly smaller (between 268 kDa and 171 kDa) in the presence of a combination of plasminogen and urokinase. The latter treatment also showed a smear of protein bands across the whole gel. The most effective digestion of soluble fibrinogen was observed after treatment with plasmin, where the native undigested fibrinogen band was replaced by a prominent band between molecular sizes of 117 kDa and 71 kDa as well as faint bands between 55 kDa and 41 kDa.*

S3: Topography of degraded fibrinogen scaffolds with planar topography

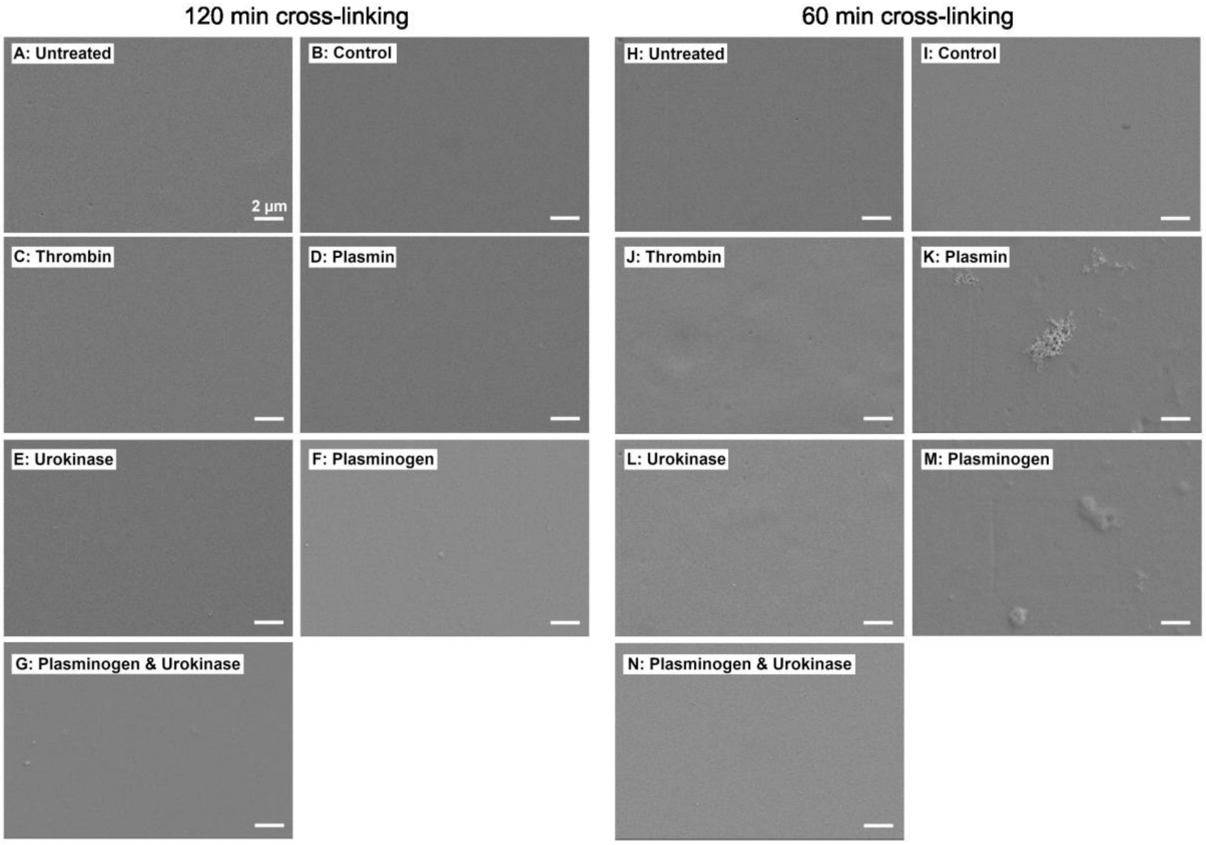

Figure S3: SEM images of degraded planar fibrinogen scaffolds. *Planar fibrinogen scaffolds were cross-linked with FA vapor for 120 min (A-G) or 60 min (H-N) and dried before incubation (untreated) or were incubated for 35 days in HEPES buffer (control) or HEPES buffer containing the respective enzymes, before being subjected to SEM imaging. The smooth topography of the scaffolds appeared to be unchanged even after 35 days of incubation in the absence or presence of different enzymes in comparison to untreated planar scaffolds without any prior treatment in an aqueous environment.*
